# Synergistic Effect of CTLA-4 Blockade and Cancer Chemotherapy in the Induction of Anti-Tumor Immunity

**DOI:** 10.1371/journal.pone.0061895

**Published:** 2013-04-23

**Authors:** W. Joost Lesterhuis, Joanne Salmons, Anna K. Nowak, Esdy N. Rozali, Andrea Khong, Ian M. Dick, Julie A. Harken, Bruce W. Robinson, Richard A. Lake

**Affiliations:** 1 National Centre for Asbestos Related Diseases, The University of Western Australia, Crawley WA, Australia and Tumour Immunology Group, School of Medicine and Pharmacology, Sir Charles Gairdner Hospital, University of Western Australia, Nedlands, Western Australia, Australia; 2 Department of Medical Oncology, Radboud University Nijmegen Medical Centre, Nijmegen, The Netherlands; 3 Department of Medical Oncology, Sir Charles Gairdner Hospital, Nedlands, Western Australia, Australia; 4 Department of Respiratory Medicine, Sir Charles Gairdner Hospital, Nedlands, Western Australia, Australia; University of Porto, Portugal

## Abstract

Several chemotherapeutics exert immunomodulatory effects. One of these is the nucleoside analogue gemcitabine, which is widely used in patients with lung cancer, ovarian cancer, breast cancer, mesothelioma and several other types of cancer, but with limited efficacy. We hypothesized that the immunopotentiating effects of this drug are partly restrained by the inhibitory T cell molecule CTLA-4 and thus could be augmented by combining it with a blocking antibody against CTLA-4, which on its own has recently shown beneficial clinical effects in the treatment of patients with metastatic melanoma. Here we show, using two non-immunogenic murine tumor models, that treatment with gemcitabine chemotherapy in combination with CTLA-4 blockade results in the induction of a potent anti-tumor immune response. Depletion experiments demonstrated that both CD4^+^ and CD8^+^ T cells are required for optimal therapeutic effect. Mice treated with the combination exhibited tumor regression and long-term protective immunity. In addition, we show that the efficacy of the combination is moderated by the timing of administration of the two agents. Our results show that immune checkpoint blockade and cytotoxic chemotherapy can have a synergistic effect in the treatment of cancer. These results provide a basis to pursue combination therapies with anti-CTLA-4 and immunopotentiating chemotherapy and have important implications for future studies in cancer patients. Since both drugs are approved for use in patients our data can be immediately translated into clinical trials.

## Introduction

Although in the past, orthodox clinical practice held that chemotherapy and immunotherapy could not be combined because of the myelosuppressive nature of most cytotoxic drugs, this notion has been challenged in recent years by a large body of experimental data (reviewed in [1], [2]). For example, treatment with anthracyclines and oxaliplatin results in immunogenic tumor cell death and platinum-based chemotherapeutics downregulate the inhibitory STAT6/PD-L2 pathway and sensitize tumor cells for T cell-mediated cytotoxicity [3]–[5]. Our group has shown that the nucleoside analog gemcitabine can enhance tumor antigen cross-presentation by dendritic cells and others have shown that this treatment leads to upregulation of tumor MHC class I expression and depletion of both regulatory T cells and myeloid-derived suppressor cells [6]–[10]. These data provide a strong rationale to exploit the immunopotentiating effect of gemcitabine by combining it with other immunotherapeutic approaches.

Immunosuppressive networks play an important role in the evasion of anti-tumor immunity, and as such could restrain the immunopotentiating effect of chemotherapy. One of the potentially relevant restraining pathways is mediated by the immune inhibitory molecule Cytotoxic T-Lymphocyte Antigen 4 (CTLA-4). The expression of CTLA-4 is upregulated following T-cell activation and the pathway has been shown to play an important immunomodulatory role in cancer. Therapeutic blockade of CTLA-4 has been shown to be an effective treatment for melanoma [11]. The anti-CTLA-4 monoclonal antibody ipilimumab is now registered by the FDA as the first treatment that has shown an overall survival benefit in a randomized phase III study in metastatic melanoma in combination with dacarbazine chemotherapy [12], [13]. However, although some patients achieved complete responses and others went on to long-term progression-free survival, the majority of patients experienced disease progression.

We set out to determine if the CTLA-4 checkpoint limits the potential therapeutic activity of gemcitabine by combining it with a CTLA-4 blocking antibody. In this study we show for the first time that CTLA-4 blockade and immunopotentiating chemotherapy in a therapeutic dose have a synergistic effect, resulting in the induction of a potent anti-tumor immune response and long-term protective immunity. In addition, we show that the overall efficacy of the combination in mice is dependent upon the timing of administration of the individual components.

## Materials and Methods

### Mice

BALB/C (H-2^d^) and C57BL/6 (H-2^b^) mice were obtained from the Animal Resources Centre (Canning Vale, Australia) and were maintained under standard conditions (M-Block Animal Facility, Queen Elizabeth II Medical Centre, The University of Western Australia). All mice used in these studies were between 8–12 weeks of age.

### Ethics Statement

All animal experiments were conducted according to The University of Western Australia Animal Ethics Committee approvals (protocol RA/3/100/1016) and the code of conduct of the National Health and Medical Research Council of Australia. The Western Australia Animal Ethics Committee specifically approved this study.

### Cell Lines

The MHC class I-positive, class II-negative, highly tumorigenic and poorly immunogenic BALB/C-derived asbestos-induced mouse mesothelioma cell line AB1, transfected with the influenza HA gene (AB1-HA) has been described before [6], [7]. For rechallenge experiments non-HA-transfected AB1 cells were used. The poorly immunogenic and highly tumorigenic Lewis Lung Cancer (LLC) cell line was obtained from CellBank Australia (Westmead NSW, Australia), where the identity of the cell line was validated. Cell lines were maintained in RPMI 1640 (Invitrogen, Mulgrave, Australia) supplemented with 20 mM HEPES, 0.05 mM 2-mercaptoethanol, 100 units/mL penicillin (CSL, Melbourne, Australia), 50 µg/mL gentamicin (David Bull Labs, Kewdale, Australia), and 10% FCS (Invitrogen). AB1-HA cells were maintained in media containing the neomycin analogue geneticin (Invitrogen) at a final concentration of 400 µg/mL. All cell lines were regularly tested and remained negative for Mycoplasma spp.

### Tumor Challenge and Experimental Protocol

ABI-HA tumor cells (1×10^6^) or LLC (2.5×10^5^) in 100 µl PBS were inoculated s.c. into the lower right flank of recipient mice. Standard chemotherapy commenced 9 days later for AB1-HA and 6 days later for LLC when a palpable tumor of approximately 10 mm^2^ was evident. Mice were injected i.p. with gemcitabine 120 µg/g body weight every third day for five doses (q3dx5), a regimen previously established as a maximal tolerated dose for BALB/C mice (Figures S1, S2 and S3) [6], [7]. Alternatively, mice were treated with a single dose of cisplatin 6 µg/g on day 9 for AB1-HA or day 6 for LLC, which we found to be the maximum tolerated dose in this model based on titration experiments (data not shown). Control mice received 100 µl PBS alone. Anti-CTLA-4 was administered i.p. every third day for four doses (q3dx4). Initially we used 100 µg per dose, but subsequent dose titration studies showed that with 75 µg per dose equal results were obtained and for that reason we took this dose for subsequent experiments (Figure S4). In combination experiments using AB1-HA with cisplatin, we used one single dose of 200 µg anti-CTLA-4 on day 9, based on a recent report demonstrating the feasibility and potency of that schedule [14], and based on our own data showing equivalency with the 75 µg q3dx4 schedule (data not shown). Tumor size was measured using micro-calipers at least three times weekly during the treatment and subsequently until tumor size reached 100 mm^2^, at which point mice were euthanized following regional animal ethics guidelines. During treatment mice weights were monitored and culled if significant weight loss (>15%) or toxicity was observed.

For some experiments mice that had shown complete regression of tumors were rechallenged with non-HA transfected AB1 mesothelioma cells in the lower left flank (Figure S5). If at least two months after rechallenge no tumors were palpable, the mice were considered to be immune. Tumor-draining lymph nodes were then collected and stained for memory T cell markers (see below). Non-tumor-bearing naïve mice were used as controls.

### Antibodies and Chemotherapy

Gemcitabine (Gemzar, Eli Lilly) was supplied by the pharmacy department of Sir Charles Gairdner Hospital. The anti-CTLA-4 (clone 9H10) monoclonal antibody was prepared and purified at the Monoclonal Antibody Facility, WAIMR (Perth, Australia). The CTLA-4 hybridoma was a kind gift from Prof. J.P. Allison (Memorial Sloan Kettering Cancer Centre, New York, US).

For depletion experiments, the following antibodies were used: anti-NK1.1 (clone PK136), anti-CD4 (clone GK1.5) and anti-CD8 (clone YTS169.4), all from the Monoclonal Antibody Facility, WAIMR (Perth, Australia). Anti-CD4 and CD8 were administered 150 µg i.v., one day before gemcitabine/anti-CTLA4, followed by 100 µg i.p. every 3 days, last dose on day 27. Anti-NK1.1 was administered 200 µg i.p on day 6, 9 and 12. Depletion was confirmed by flow cytometry of peripheral blood from tail bleeds (Figure S6).

The following antibodies were used for flow cytometry: CD3 FITC, CD4-PECy7, CD4 Pac Blue, CD8 PerCpCy5.5, CD3 PE and ICOS APC, CD44-PE, CD49b FITC, CD62L-FITC, (all Biolegend), Ki67 AF488, Ki 67 PE and CD4 APCH7 (all BD Bioscience), CD3 PeCy7 FoxP3-PerCPCy5.5 and CD8 PECy7, CD8 ef780 (eBioscience).

### Cell Staining and Flow Cytometry Analysis

Peripheral blood sampling was performed via tail bleeds on day 29. A volume of <100 µl of blood was collected in a heparin tube. Antibody cocktails of surface stains (CD3, CD4, CD8 and ICOS) were prepared and 20 µl added to 30 µl blood for 1 hour. Samples were lysed (BD FACS lysing solution) and permeabilized (eBioscience Fixation/Perm Buffer), the antibody for intracellular staining (Ki-67) was prepared and 20 µl added for 45 mins. Samples were resuspended in 200 µl stabilizing fixative (BD) and 50000 lymphocyte gated events were acquired on the FACS Canto II flow cytometer (BD Biosciences) and data were analysed using FlowJo software.

For some experiments, involving mice that had been cured with treatment and subsequently resisted a rechallenge of tumor cells on the contralateral flank, tumor-draining lymph nodes (TDLN) were harvested (see above) for analysis of T memory cell subsets. Lymph nodes from both flanks were harvested and pooled and stained for CD4, CD8, CD44 and CD62L, according to the same protocol as the flow cytometry analysis of peripheral blood (see above and Figure S5).

For analysis of T cell responses in the tumor, TDLN (ipsilateral axillary and inguinal nodes) and spleen, mice were culled on day 15 and the organs were harvested. Day 15 was chosen as time point since from approximately day 12 the growth curves between the groups started to divide, allowing adequate evaluation of T cell responses. Spleens and LNs were mashed between glass slides, resuspended in red blood cell lysis solution (eBioscience) and filtered through a 40 µm filter (BD) and stained with the relevant antibodies. Tumors were minced finely and transferred to digestion solution consisting of RPMI/2% FCS with 10 mg/ml Collagenase and 1 mg/ml DNAse I (Sigma-Aldrich) and incubated for 1 hour on a roller bank. During the last 10 minutes EDTA was added to a final solution of 5 mM. Samples were washed with RPMI/2%FCS and filtered through a 40 µm filter and stained with the relevant antibodies.

### Statistical Analyses

Data were analyzed using Prism 4.0 (GraphPad Software, Inc.). Tumor growth data were analyzed using the PASW statistics version 18 MIXED procedure (IBM SPSS, Chicago IL). Comparisons between treatment groups at each time point were adjusted for multiple comparisons by the Sidak method. Data for tumor survival were analyzed according to the Kaplan Meier method and survival proportions were compared between groups using a Log Rank Test. Data from T cell subsets were compared with the Student’s t test. Differences were considered significant when the P value was <0.05.

## Results

### Anti-CTLA-4 and Gemcitabine Combine in a Therapeutically Synergistic Manner

**Figure 1:**
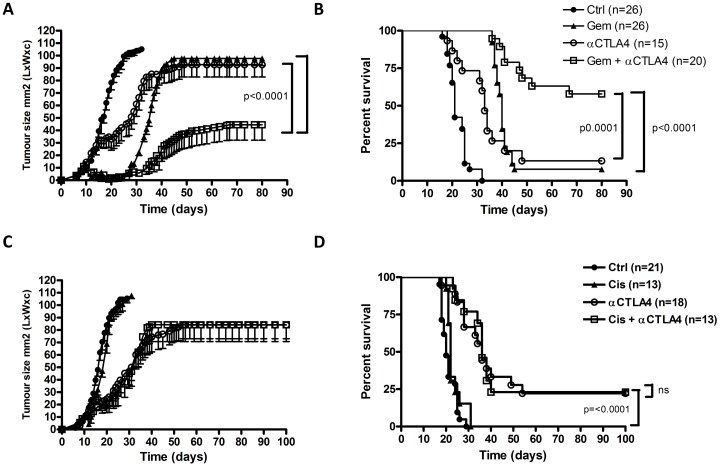
Combination of CTLA-4 blockade and gemcitabine chemotherapy results in synergistic anti-tumor effect. (A) Tumor surface in mm^2^ (mean ± SD) of AB1-HA tumors that were injected on day 0, mice (n = 87) were treated on day 9/12/15/18 with 75 µg anti-CTLA-4 and with 120 µg/g gemcitabine on day 9/12/15/18/21, or with PBS (pooled data of 5 separate experiments are shown). (B) Kaplan-Meier survival plot of the same experiment. (C) Tumor surface in mm^2^ (mean ± SD) of AB1-HA tumors that were injected on day 0, mice (n = 65) were treated on day 9 with 200 µg anti-CTLA-4 and 6 µg/g cisplatin, or with PBS (pooled data from 3 separate experiments are shown). (D) Kaplan-Meier survival plot of the same experiment.

Building on previous data demonstrating gemcitabine as an immunogenic cytotoxic drug [7], we hypothesized that the therapeutic efficacy of gemcitabine could be further enhanced by combining it with a blocking antibody against CTLA-4. AB1-HA-inoculated BALB/C mice were treated with anti-CTLA-4 in combination with gemcitabine (Figure S1 and Figure 1). Treatment with gemcitabine alone resulted in good control of tumor outgrowth when the drug was administered, as previously reported, however tumor progressed on cessation of treatment in the majority of mice [15]. Treatment with anti-CTLA-4 alone reduced the rate of tumor growth but was less effective than gemcitabine as a monotherapy (Figure 1A). However, when anti-CTLA-4 and gemcitabine were combined, a clear additive effect of both treatments with a significant delay of tumor outgrowth was observed. The number of animals that achieved complete regression was superadditive (∼60% in the combination group versus ∼13% for anti-CTLA-4 and ∼8% for gemcitabine alone in the AB1-HA model, Figs. 1A and B). We also found enhanced tumor control in the LLC model, although the effect was less pronounced (Figure S7). This accords with human studies using immune checkpoint blocking antibodies, demonstrating major differences in efficacy between different cancer types [16]. Interestingly, when we treated the mice with the non-immunogenic chemotherapeutic drug cisplatin [17], there was no clear synergistic effect in either model (Figure 1C and D and Figure S7).

**Figure 2:**
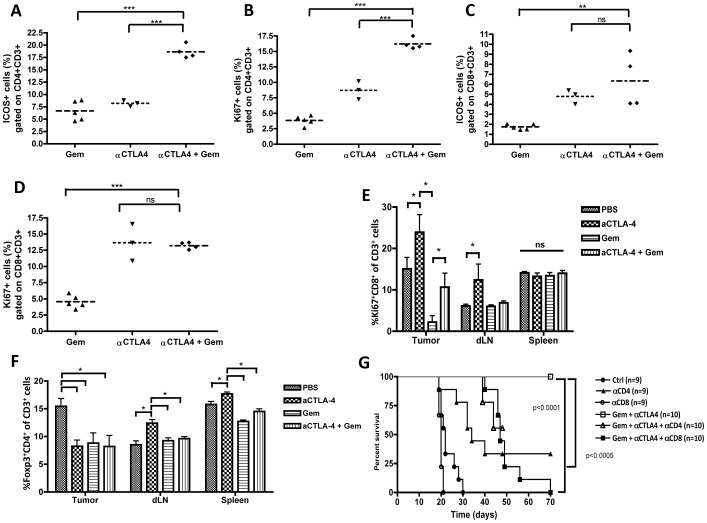
Combination of CTLA-4 blockade and gemcitabine chemotherapy results in enhanced T cell activation and proliferation and is dependent on CD4+ and CD8+ T cells. A comparison is shown of peripheral blood T cell activation and proliferation markers on day 29 after inoculation for the different treatment groups (*p≤0.05; **p<0.01***p<0.001). ICOS^+^/CD4^+^ Th cells (A); Ki-67^+^/CD4^+^ Th cells (B); CD8^+^/ICOS^+^ CTLs(C) and CD8^+^/Ki-67^+^ CTLs (D). (E and F) Flow cytometric analysis of proliferating CD8^+^ T cells and Treg in tumor, tumor-draining lymph node and spleen on day 15. Depicted are the percentage of Ki-67^+^CD8^+^ of CD3^+^ cells and Foxp3^+^CD4^+^ of CD3^+^ cells (F). Six mice per group were tested for control and anti-CTLA-4, 12 mice per group for gemcitabine-containing regimes pooled per 2 mice because of the small tumor size in these groups. Means with SEMs are shown (n = 36). (G) Kaplan-Meier survival plot of AB1-HA tumors that were injected on day 0, mice (n = 57) were treated with anti-CTLA-4 and/or gemcitabine, or with PBS in combination with depleting antibodies against CD4 or CD8 (pooled data of 2 separate experiments are shown).

Previous studies in cancer patients and animals have suggested that ICOS^+^ T cells play an important role in the action of anti-CTLA-4, as well as having prognostic significance [18], [19]. We analyzed ICOS expression and proliferative status of circulating T cells in the mice and found that mice that were treated with the combination therapy showed a significant increase in CD4^+^ICOS^+^ T cells in peripheral blood, as well as a clear increase in CD4^+^ proliferative T cells as determined by Ki-67 staining (Figure 2A–D, p<0.001).

To gain more insight into the composition of tumor-infiltrating cells during treatment, we calculated the frequency of Foxp3+CD4+ Tregs, CD49b^+^CD3^−^ NK cells and ICOS^+^CD4^+^ activated Th cells and Ki-67^+^CD8^+^ proliferating CTLs in tumor, tumor-draining lymph nodes (TDLN) and spleen on day 15 (Figure 2E–F, Figure S8). The percentage of CD8^+^ CTLs did not differ between treatment groups (Figure S8), but their proliferative capacity, as measured by Ki-67 did increase when mice were treated with anti-CTLA4, both in tumor and TDLN. Interestingly, the relative loss of proliferating tumor-infiltrating CD8^+^ T cells in gemcitabine-treated mice was partly rescued by anti-CTLA-4 (Figure 2E). Tumor CD4+ T cell infiltration was not significantly altered by either gemcitabine or anti-CTLA-4, although ICOS expression as a marker of activation was decreased in all gemcitabine-treated mice, either with or without anti-CTLA4 (Figure S8). The percentage of tumor-infiltrating Foxp3^+^CD4^+^ T cells was significantly decreased in tumors treated with gemcitabine, anti-CTLA-4 or the combination treatment (Figure 2F), a finding consistent with previously published data [10]. No clear differences were observed in NK cell numbers between treatment groups (Figure S8).

To investigate whether the enhanced response to the combination therapy involved mainly CD4^+^ or CD8^+^ T cells or NK cells we performed depleting experiments using monoclonal antibodies against CD4, CD8 (AB1-HA model) and NK1.1 (LLC model, since BALB/C mice do not express NK1.1). We found that the therapeutic effect of gemcitabine plus anti-CTLA4 was completely abrogated when either CD4^+^ or CD8^+^ cells were depleted (Figure 2G), whereas depletion of NK cells did not affect the efficacy of the treatment (Figure S9). Taken together, these data demonstrate that anti-CTLA-4 and chemotherapy synergize in the induction of a potent anti-tumor immune response, with an important role for both CD4^+^ and CD8^+^ T cells for optimal therapeutic effect.

### Anti-CTLA-4 and Gemcitabine Combination Therapy Induces Long-lasting Protective Anti-tumor Immunological Memory

**Figure 3:**
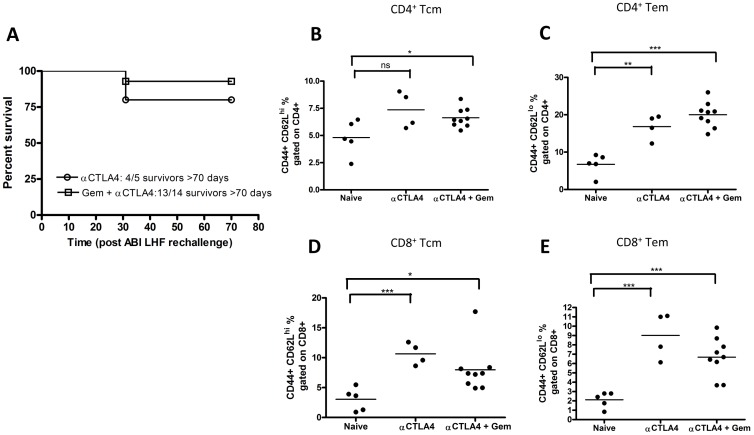
Combination of CTLA-4 blockade and gemcitabine chemotherapy results in the induction of protective T cell memory. (A) Kaplan-Meier survival plot of mice that had been cured by either anti-CTLA-4 alone or combination therapy and that were subsequently rechallenged with AB1 mesothelioma cells, showing protective immunity in 80% and 92% respectively. T cell subset analysis in tumor-draining lymph nodes in these mice (*p<0.05; **p<0.01***p<0.001): CD44^+^/CD62L^+^/CD4^+^ T central memory cells (B); CD44^+^/CD62L^−/^CD4^+^ T effector memory cells (C); CD44^+^/CD62L^+^/CD8^+^ T central memory cells (D); CD44^+^/CD62L^−/^CD8^+^ T effector memory cells (E).

One of the important theoretical advantages of immunotherapy over chemotherapy is that the former has the potential to induce immunological memory and therefore the potential to achieve durable responses. We tested whether combination treatment with anti-CTLA-4 and gemcitabine resulted in anti-tumor immunological memory. We reinoculated mice that had completely rejected their tumors following combination treatment and found that 93% (13 out 14 mice) of these mice were completely resistant to tumor rechallenge (Figure 3A). Importantly, for rechallenge experiments we used AB1 cells that were not transfected with HA, indicating that the induced immunity was against shared tumor antigens on the AB1 mesothelioma cells and not solely against the transfected HA antigen. Flow cytometric analysis of T cell subsets in the draining lymph nodes of these mice showed increased levels of both central memory and effector memory CD4^+^ T cells, and to a lesser extent CD8^+^ memory cells (Figure 3B–E, p<0.001). Together, these data suggest that the combination treatment results in an increase of memory T cells and the induction of protective immunity.

### Efficacy of Anti-CTLA-4/Gemcitabine Depends on Timing

**Figure 4:**
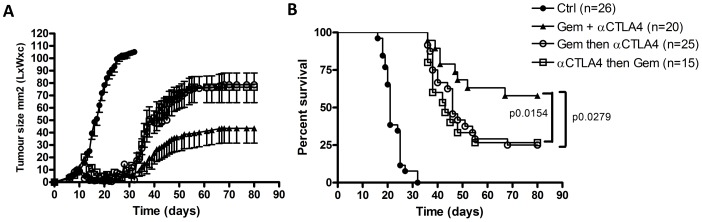
The efficacy of combining CTLA-4 blockade with gemcitabine critically depends on timing. (A) Tumor area in mm^2^ (mean ± SD) of AB1-HA tumors that were injected on day 0, mice (n = 86) were treated with different schedules of anti-CTLA4 and gemcitabine (see Figure S2), or with PBS (pooled data of 3 separate experiments are shown). (B) Kaplan-Meier survival plot of the same experiment.

In order to determine the optimal treatment schedule in terms of timing of both anti-CTLA-4 and gemcitabine, we treated AB1-HA tumor-bearing mice with three different regimes: gemcitabine followed by anti-CTLA-4, concomitant combination therapy, and anti-CTLA-4 followed by gemcitabine (Figure S3; Figure 4). One animal in the anti-CTLA-4 followed by gemcitabine group was culled because of weight loss greater than 15%, otherwise there was no apparent toxicity. We observed marked differences in tumor outgrowth between these groups (Figure 4). There was no significant additive value of the combination therapy over either anti-CTLA-4 or gemcitabine alone when the chemotherapeutic drug was administered separately from anti-CTLA-4. The synergistic anti-tumor effect was only observed when the both drugs were given concomitantly. Surprisingly, when only the first dose of gemcitabine was omitted (as in the ‘anti-CTLA-4 first’ arm versus the concomitant arm), the anti-tumor effect decreased dramatically (Figure 4). These data show that appropriate scheduling of the separate compounds is critical for optimal efficacy.

## Discussion

The combination of chemotherapy and immunotherapy in the treatment of cancer holds unrealized promise [1]. The recently FDA-approved anti-CTLA-4 antibody is a logical and easily translatable immunotherapeutic approach to combine with chemotherapy. We hypothesized that we would find a synergistic interaction with a combination of anti-CTLA-4 blockade and an immunopotentiating cytotoxic drug. We anticipated that the chemotherapy would cause tumor shrinkage and immunogenic antigen release while the anti-CTLA-4 would enhance T cell activation and expansion. Prior data to support this hypothesis were limited. A large phase III trial in metastatic melanoma comparing anti-CTLA-4 plus DTIC versus DTIC alone found a survival benefit for the combination therapy compared to DTIC chemotherapy alone [13]. But because there was no comparison with anti-CTLA-4 alone, the relative contribution of the chemotherapy to the observed effect could not be accurately assessed. Similarly, a phase II study in non-small cell lung cancer, found improved progression-free survival for combination of ipilimumab and chemotherapy versus chemotherapy alone; again here ipilimumab alone was not a comparator [20]. In a phase II study that did compare ipilimumab alone versus ipilimumab plus DTIC, but using lower doses of study drug, there was a trend towards better disease control rate for the combination arm, but this did not reach significance [21]. Based on these published human studies, no definitive conclusion can be drawn on a possible synergistic effect of anti-CTLA-4 and chemotherapy. Although a previous animal study did find enhanced anti-tumor efficacy when anti-CTLA4 was added to melphalan chemotherapy, this experiment used a subtherapeutic dose of melphalan, intended to skew T cell responses towards a Th1 phenotype [22]. Recently, Wu and colleagues found that anti-CTLA-4 treatment in combination with cisplatin resulted in better disease control in a murine mesothelioma model, when tumors were treated before they were palpable, presumably due to inhibited cancer cell repopulation [23]. We found no published animal data relevant to our hypothesis, using therapeutic dosages of chemotherapy in overt cancer.

As gemcitabine is widely used in the treatment of many cancer types, including mesothelioma, we tested the combination in a well–established non-immunogenic murine model of mesothelioma. Treatment of AB1-HA with gemcitabine results in moderate tumor reduction or delayed tumor outgrowth in this model, thereby mimicking the clinical situation in the chemotherapeutic treatment of most metastatic cancers.

We found here that combination therapy of gemcitabine and anti-CTLA-4 exerted a far greater anti-tumor effect than either of the agents alone, thus acting in a synergistic manner (Figure 1). This correlated with a pronounced increase in CD4^+^ICOS^+^ T cells in peripheral blood, as well as a clear increase in proliferating CD4^+^ T cells as determined by Ki-67 staining, although we did not detect this increase in the tumor as well (Figure 2). CD4^+^ T cell infiltration in the tumor was enhanced by the combination treatment, and a gemcitabine-associated decrease in proliferating tumor-infiltrating CD8^+^ T cells was partly rescued by CTLA-4 blockade. Importantly, we did not find any reduction in tumor growth when anti-CTLA-4 was combined with cisplatin. Cisplatin has been shown to induce a non-immunogenic form of cell death [17], and although it does downregulate the inhibitory molecule PD-L2 [5], the tumor model we use expresses only very low levels of PD-L2 (data not shown). Therefore, we consider cisplatin to be a non-immunopotentiating form of chemotherapy in this model. These results suggest that combination treatment with anti-CTLA-4 will be most potent when combined with immunopotentiating chemotherapy.

Since one of the theoretical advantages of combining chemotherapy with immunotherapy is the induction of a long-lasting immunological memory, we investigated the memory T cell response in mice with tumors that had regressed upon treatment (Figure 3). We found that these mice had enhanced levels of both CD4^+^ and CD8^+^ effector memory and central memory T cells in the tumor-draining lymph nodes, correlating with protective immunity to a rechallenge with tumor cells. These findings accord with studies in a murine OVA-expressing *Listeria monocytogenes* model, in which CD8^+^ T cell memory was enhanced by a single dose of anti-CTLA-4 [14]. Importantly, in our model, neither the formation of CD4^+^ nor CD8^+^ memory T cells was hampered by gemcitabine.

Our third aim was to determine the optimal sequence of chemotherapy and anti-CTLA-4 therapy. Since it is known from several animal studies that timing is crucial in the use of anti-CTLA-4 when combined with vaccination approaches [24], [25], we hypothesized that optimal timing/scheduling in combination with chemotherapy would also be critical for anti-CTLA-4 efficacy. We found that the efficacy of the combination indeed depended on scheduling: if gemcitabine was administered before or after anti-CTLA-4, there was no additive value above either therapy alone, whereas concomitant treatment did result in disease control in the majority of mice (Figure 4).

In conclusion, our results demonstrate that anti-CTLA-4 therapy and cytotoxic chemotherapy can have a clear synergistic effect in the treatment of cancer. Our data provide a rationale to further develop combinations of cytotoxic drugs and anti-CTLA-4 in the clinic. However, based on our data we suggest that for different groups of cytotoxic anti-cancer compounds, their optimal schedule and immunogenicity should first be carefully determined in pre-clinical models and small clinical studies.

## Supporting Information

Figure S1 **Treatment schedule of gemcitabine and anti-CTLA-4 in the AB1-HA model.** Balb/c mice were inoculated with 1×10^6^ AB1-HA murine mesothelioma cells on day 0 and subsequently injected i.p with PBS, 120 µg/g body weight gemcitabine every third day for five doses (q3dx5) on days 9–12–15–18–21 or 75 µg anti-CTLA-4 (q3dx4) on days 9–12–15–18, either alone or in combination, as indicated.
(PDF)

Figure S2 **Treatment schedule of gemcitabine and anti-CTLA-4 in the LLC model.** C57BL/6 mice were inoculated with 2.5×10^5^ LLC murine lung cancer cells on day 0 and subsequently injected i.p with PBS, 120 µg/g body weight gemcitabine every third day for five doses (q3dx5) on days 6–9–12–15–18 or 75 µg anti-CTLA-4 (q3dx4) on days 6–9–12–15, either alone or in combination, as indicated.
(PDF)

Figure S3 **Treatment schedule of combination therapy of gemcitabine and anti-CTLA-4 in the AB1-HA model, comparing different treatment schedules.** Balb/c mice were inoculated with 1×10^6^ AB1-HA murine mesothelioma cells on day 0 and subsequently injected i.p with 120 µg/g body weight gemcitabine (q3dx5) and 75 µg anti-CTLA-4 (q3dx4) divided over three groups, ‘concurrent’ (anti-CTLA-4 on days 9–12–15–18; gemcitabine on days 9–12–15–18–21), ‘anti-CTLA-4 first’ (anti-CTLA-4 on days 9–12–15–18; gemcitabine on days 12–15–18–21–24) and ‘gemcitabine first’ (gemcitabine on days 9–12–15–18–21; anti-CTLA-4 on days 24–27–30–33).
(PDF)

Figure S4 **Dose-optimisation study of anti-CTLA4 in the AB1-HA model.** Tumor surface in mm^2^ (mean ± SD) of AB1-HA tumors that were injected on day 0, mice (n = 40) were treated with 75 µg anti-CTLA-4 i.p. on days 9–12–15–18 in the indicated dosages and with gemcitabine 120 µg/g body weight on days 12–15–18–21–24.
(PDF)

Figure S5 **Gating strategy for determination of memory T cell subsets in tumor-draining lymph nodes, using flow cytometry.** Tumor-draining lymph nodes were harvested as described in the materials and methods section. Based on forward and side scatter, populations enriched for lymphocytes were gated, from which either CD4-PeCy7 positive or CD8-APC positive cells were gated. Within these populations, the CD62L-FITC and CD44-PE fluorescence signal were determined. Central memory T cells were defined as CD44^+^/CD62L^hi^, effector memory T cells were defined as CD44^+^/CD62L^lo^.
(PDF)

Figure S6 **Verification of depletion of CTL/Th/NK cells.** Mice were treated with αCD4/αCD8 (q3,dx7), starting on day 8 with 150 µg i.v, followed by 100 µg i.p on days 11, 14, 17, 20, 23, 26. Representative peripheral tail bleeds on day 19 are shown. Mice were treated with anti-NK1.1 (q3,dx3) starting on day 6 with 150 µg i.v, followed by 200 µg i.p on days 9 and 12. Representative peripheral tail bleeds on day 11 are shown.
(PDF)

Figure S7 **Effect of combination treatment on tumor outgrowth with chemotherapy and anti-CTLA-4 in the LLC model.** Tumor surface in mm^2^ (mean ± SD) of LLC tumors that were injected on day 0, mice (n = 57) were treated with anti-CTLA-4 and/or gemcitabine or cisplatin. A representative of 3 separate experiments is shown (n = 30). The difference in tumor outgrowth was significantly less for the combination treatment from day 13 on when compared with anti-CTLA-4 alone and from day 18 on when compared with gemcitabine alone (p<0.05).
(PDF)

Figure S8 **Frequencies of CD4+ Th cells, CD8+ CTLs, CD49b+CD3- NK cells and ICOS+CD4+ activated Th cells in tumor, tumor-draining lymph nodes (TDLN) and spleen.** Populations were measured on day 15 (n = 36, 6 mice per group for control and anti-CTLA-4, 12 mice per group for gemcitabine-containing regimes pooled per 2 mice because of the small tumor size in that groups), means with SEMs are shown (*p<0.05).
(PDF)

Figure S9 **The effect of NK-depletion on the efficacy of gemcitabine and anti-CTLA-4 in the LLC model.** Tumor surface in mm^2^ (mean ± SD) of LLC tumors that were injected on day 0, mice (n = 57) were treated with anti-CTLA-4 and/or gemcitabine in combination with an anti-NK1.1 depleting antibody. A representative of 2 separate experiments is shown (n = 20). Mice were treated with anti-NK1.1 (q3,dx3) starting on day 6 with 150 µg i.v, followed by 200 µg i.p on days 9 and 12. Anti-CTLA4 (q3,dx4) was administered 75 µg i.p on days 9, 12, 15, 18 and gemcitabine (q3,dx5) 120 µg/g i.p on days 9, 12, 15, 18, 21. NK depletion did not change the anti-tumor effect of combination treatment with anti-CTLA-4 and gemcitabine.
(PDF)
