# Supplementary figures and images for: Synergistic Effect of CTLA-4 Blockade and Cancer Chemotherapy in the Induction of Anti-Tumor Immunity
Source: PLoS One. 2013 Apr 23;8(4):e61895. doi: 10.1371/journal.pone.0061895 (PMC3633941; doi:10.1371/journal.pone.0061895)

Figure S1

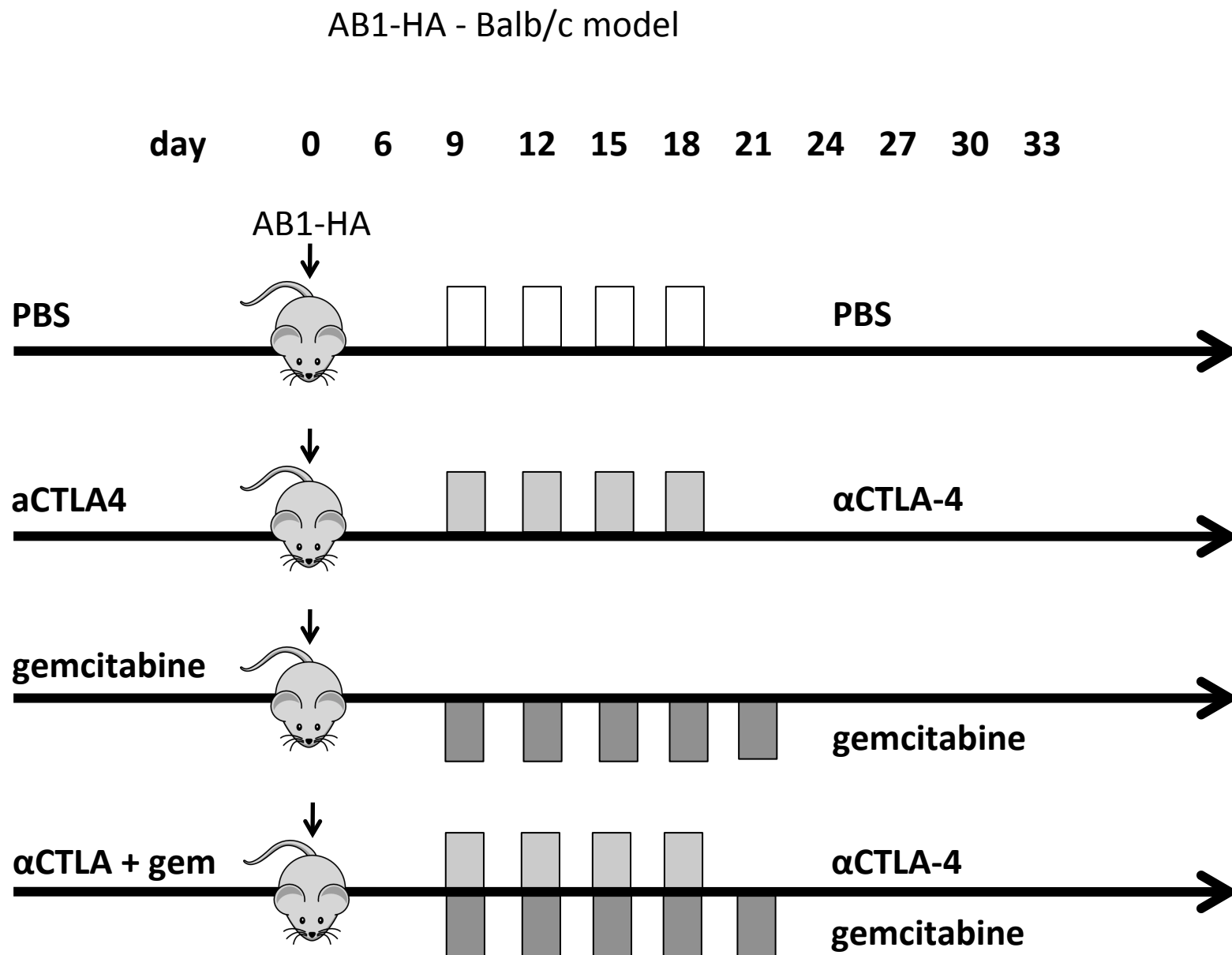

Supplement: Figure S1 — Treatment schedule of gemcitabine and anti-CTLA-4 in the AB1-HA model. Balb/c mice were inoculated with 1×10^6^ AB1-HA murine mesothelioma cells on day 0 and subsequently injected i.p with PBS, 120 µg/g body weight gemcitabine every third day for five doses (q3dx5) on days 9–12–15–18–21 or 75 µg anti-CTLA-4 (q3dx4) on days 9–12–15–18, either alone or in combination, as indicated. (PDF) [file pone.0061895.s001.pdf]

Figure S2

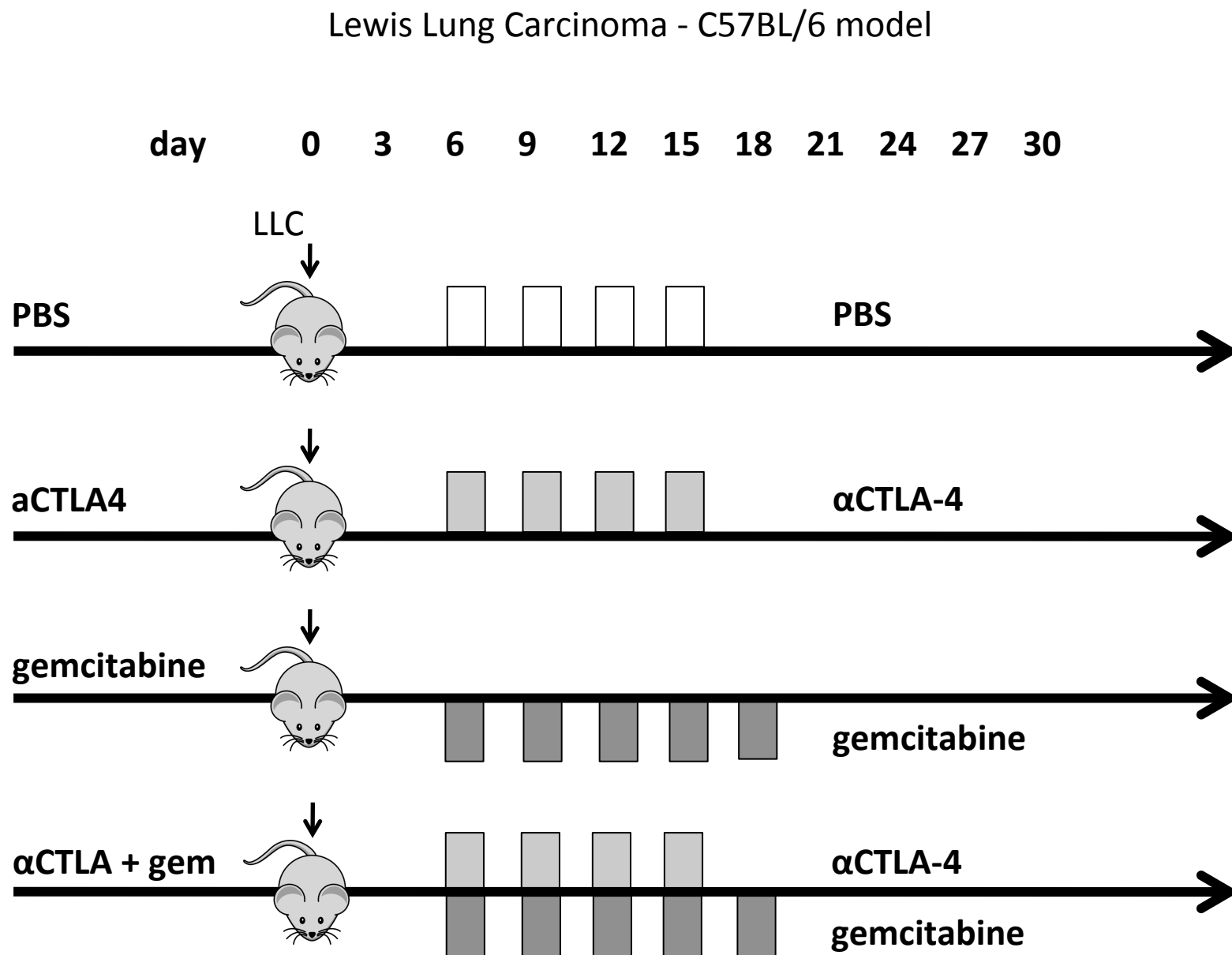

Supplement: Figure S2 — Treatment schedule of gemcitabine and anti-CTLA-4 in the LLC model. C57BL/6 mice were inoculated with 2.5×10^5^ LLC murine lung cancer cells on day 0 and subsequently injected i.p with PBS, 120 µg/g body weight gemcitabine every third day for five doses (q3dx5) on days 6–9–12–15–18 or 75 µg anti-CTLA-4 (q3dx4) on days 6–9–12–15, either alone or in combination, as indicated. (PDF) [file pone.0061895.s002.pdf]

Figure S3

### The influence of scheduling in the AB1-HA - Balb/c model

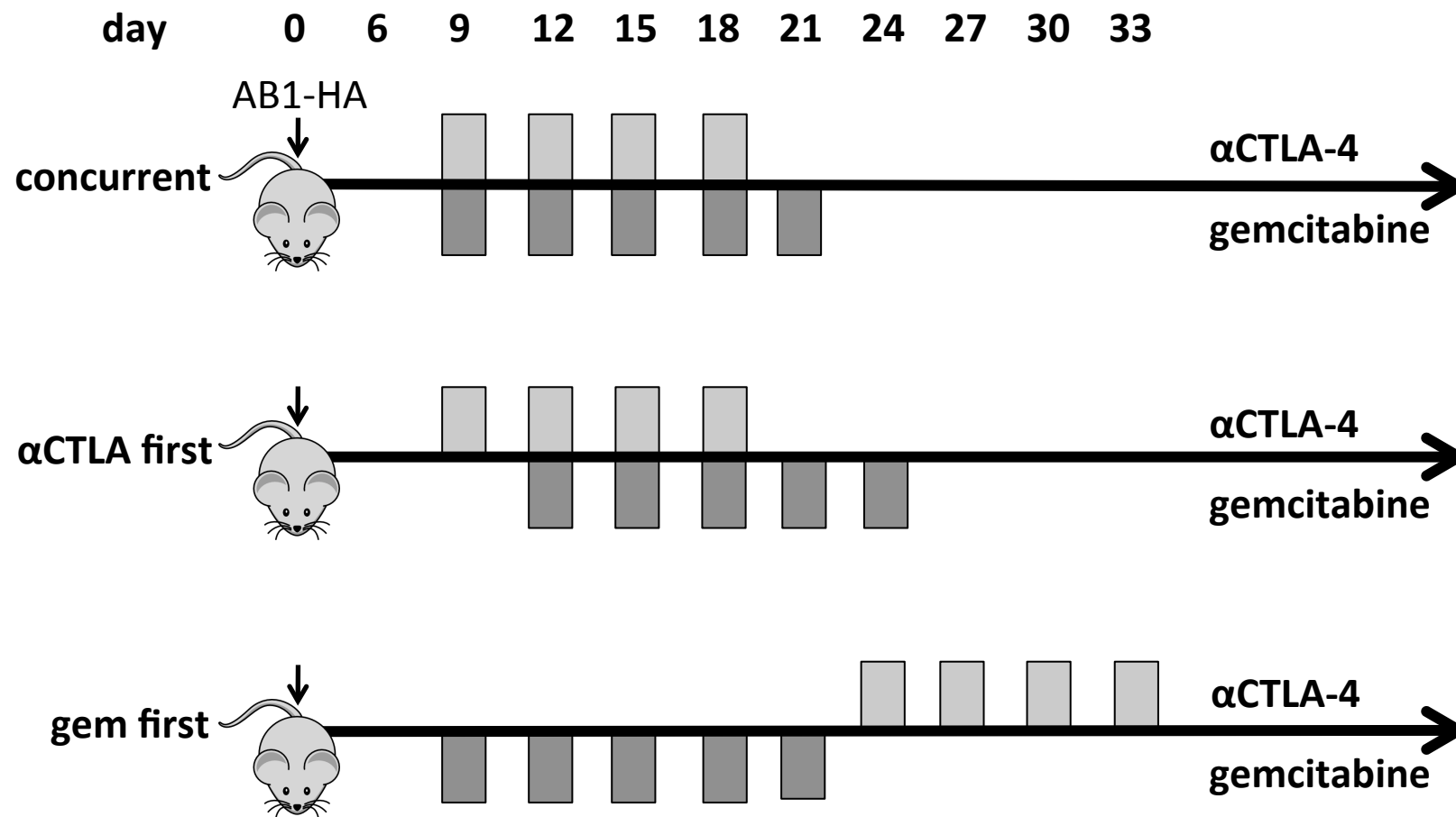

Supplement: Figure S3 — Treatment schedule of combination therapy of gemcitabine and anti-CTLA-4 in the AB1-HA model, comparing different treatment schedules. Balb/c mice were inoculated with 1×10^6^ AB1-HA murine mesothelioma cells on day 0 and subsequently injected i.p with 120 µg/g body weight gemcitabine (q3dx5) and 75 µg anti-CTLA-4 (q3dx4) divided over three groups, ‘concurrent’ (anti-CTLA-4 on days 9–12–15–18; gemcitabine on days 9–12–15–18–21), ‘anti-CTLA-4 first’ (anti-CTLA-4 on days 9–12–15–18; gemcitabine on days 12–15–18–21–24) and ‘gemcitabine first’ (gemcitabine on days 9–12–15–18–21; anti-CTLA-4 on days 24–27–30–33). (PDF) [file pone.0061895.s003.pdf]

Figure S4

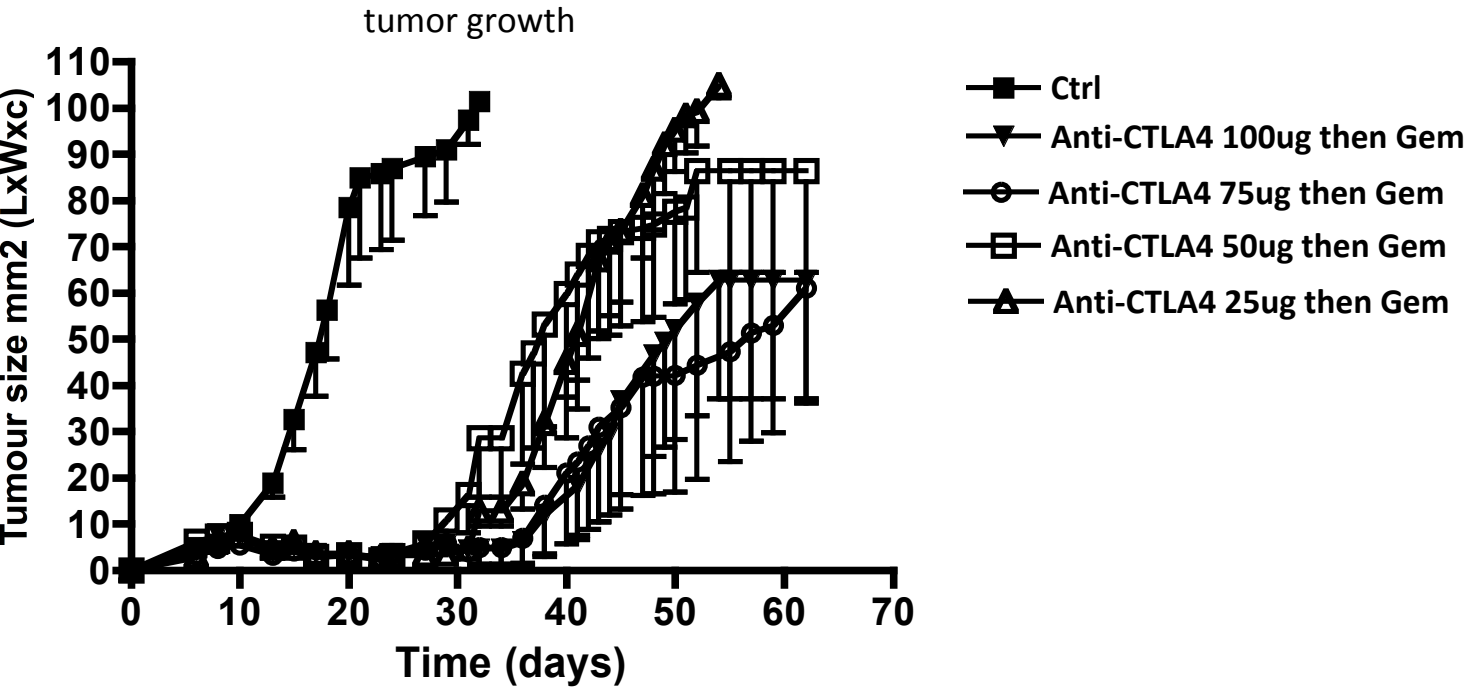

Supplement: Figure S4 — Dose-optimisation study of anti-CTLA4 in the AB1-HA model. Tumor surface in mm2 (mean ± SD) of AB1-HA tumors that were injected on day 0, mice (n = 40) were treated with 75 µg anti-CTLA-4 i.p. on days 9–12–15–18 in the indicated dosages and with gemcitabine 120 µg/g body weight on days 12–15–18–21–24. (PDF) [file pone.0061895.s004.pdf]

Figure S5

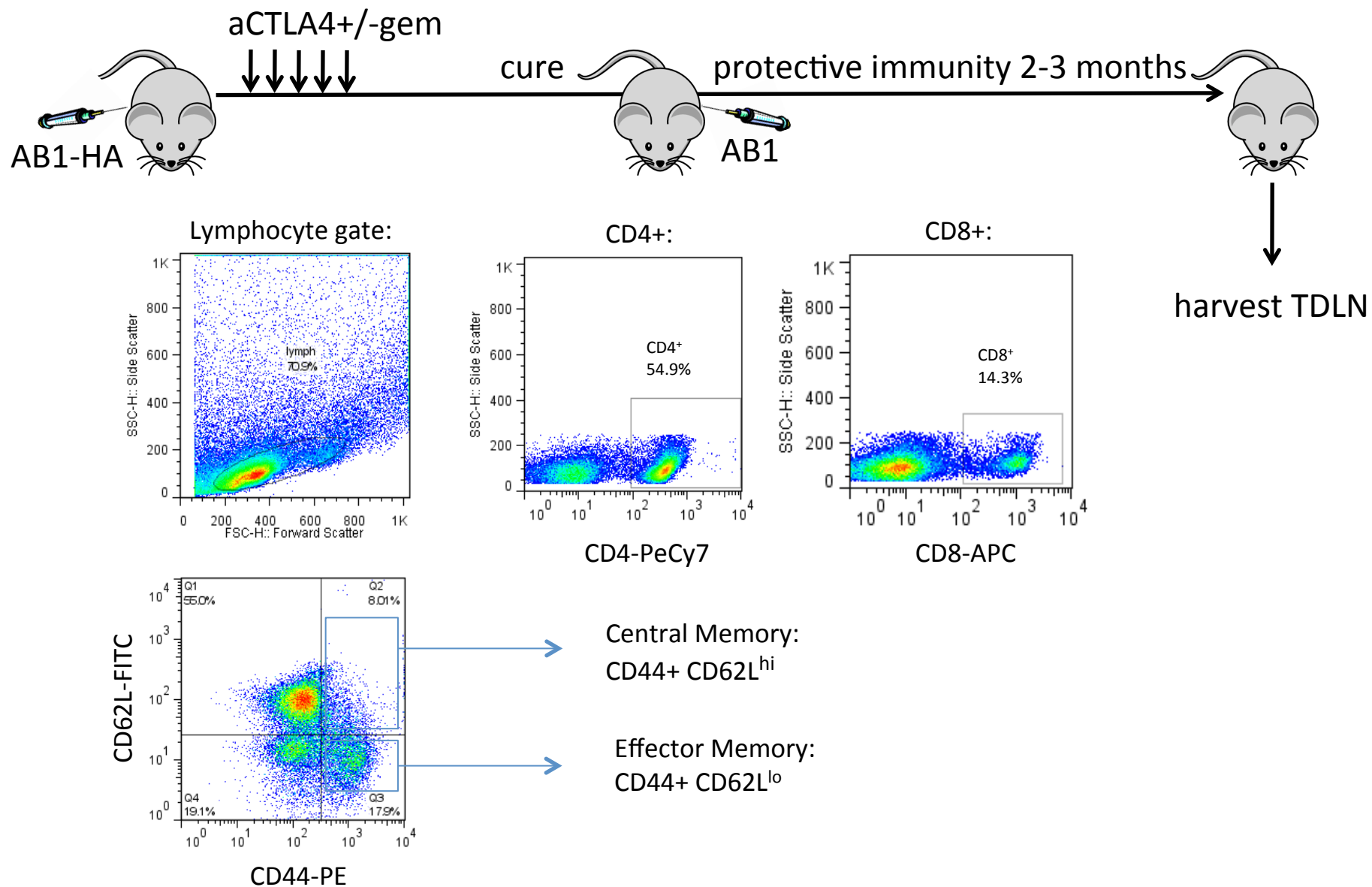

Supplement: Figure S5 — Gating strategy for determination of memory T cell subsets in tumor-draining lymph nodes, using flow cytometry. Tumor-draining lymph nodes were harvested as described in the materials and methods section. Based on forward and side scatter, populations enriched for lymphocytes were gated, from which either CD4-PeCy7 positive or CD8-APC positive cells were gated. Within these populations, the CD62L-FITC and CD44-PE fluorescence signal were determined. Central memory T cells were defined as CD44+/CD62Lhi, effector memory T cells were defined as CD44+/CD62Llo. (PDF) [file pone.0061895.s005.pdf]

Figure S6

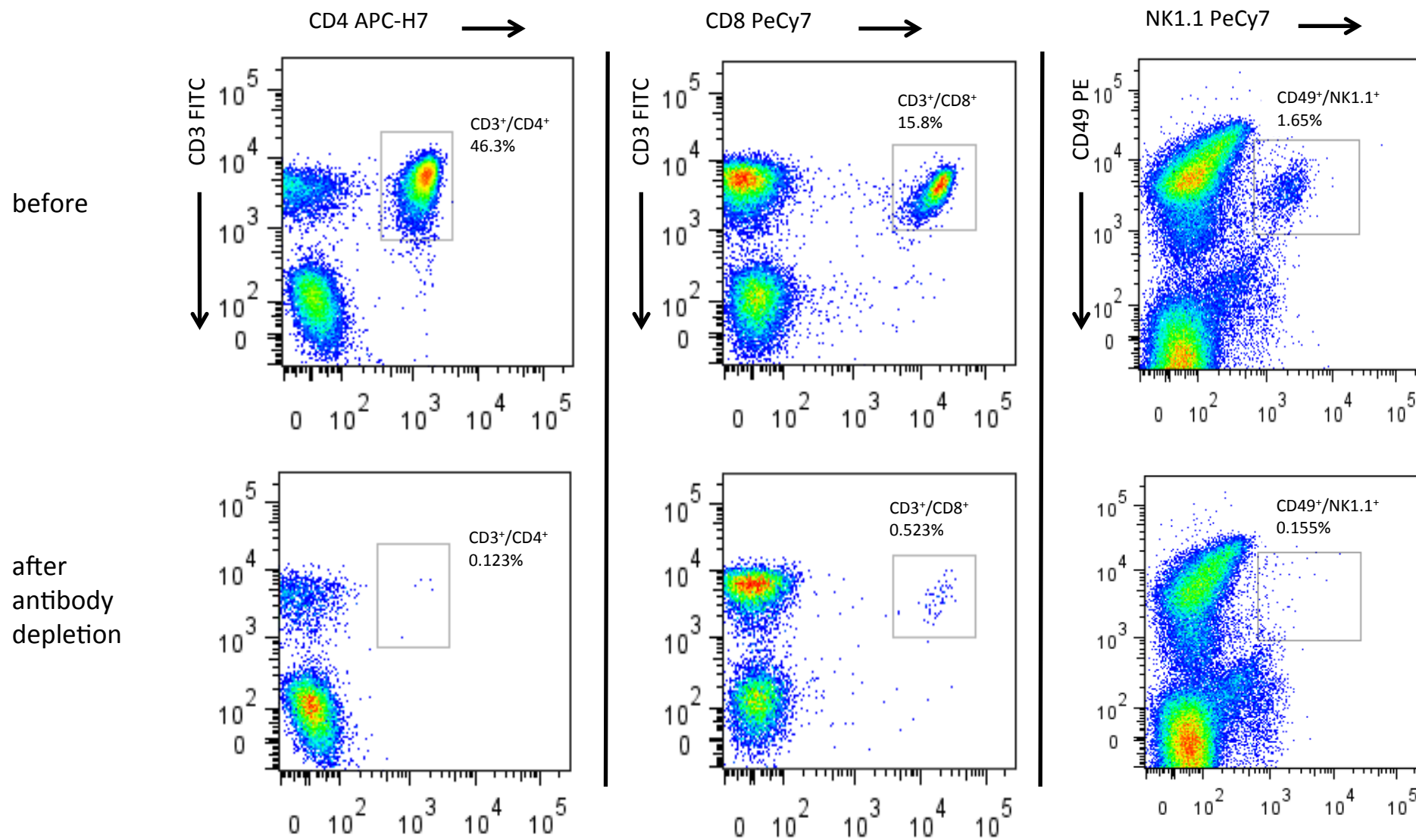

Supplement: Figure S6 — Verification of depletion of CTL/Th/NK cells. Mice were treated with αCD4/αCD8 (q3,dx7), starting on day 8 with 150 µg i.v, followed by 100 µg i.p on days 11, 14, 17, 20, 23, 26. Representative peripheral tail bleeds on day 19 are shown. Mice were treated with anti-NK1.1 (q3,dx3) starting on day 6 with 150 µg i.v, followed by 200 µg i.p on days 9 and 12. Representative peripheral tail bleeds on day 11 are shown. (PDF) [file pone.0061895.s006.pdf]

Figure S7

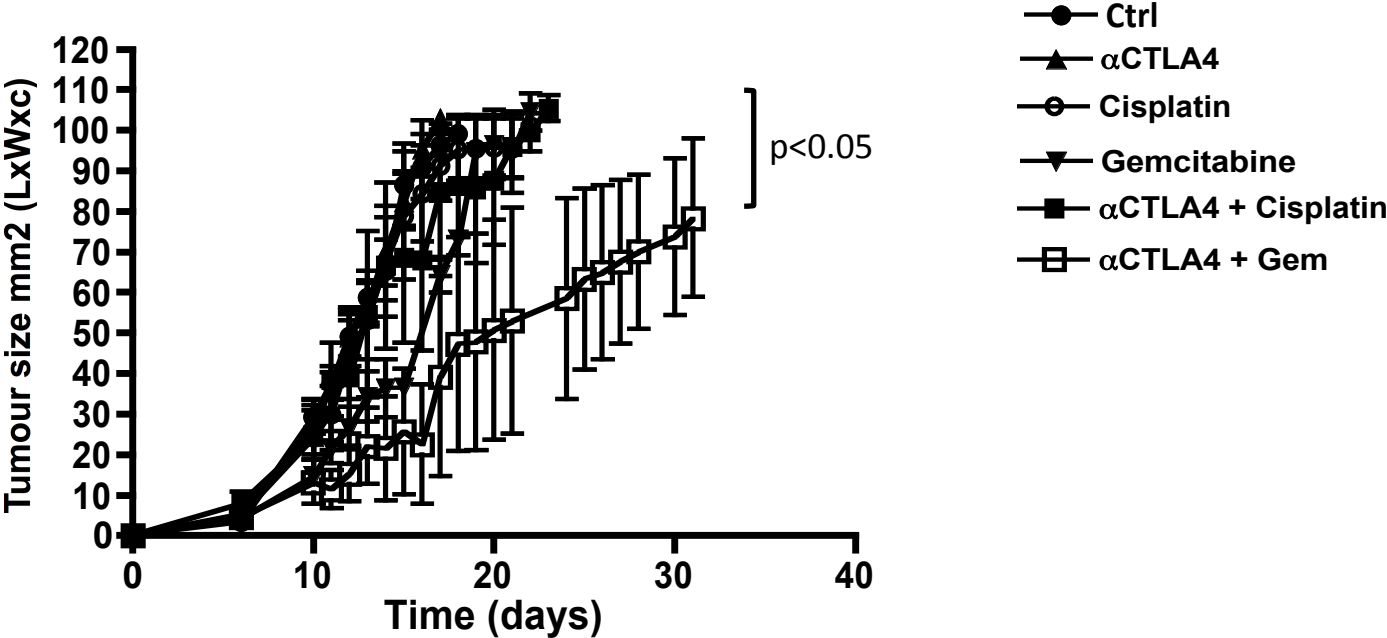

Supplement: Figure S7 — Effect of combination treatment on tumor outgrowth with chemotherapy and anti-CTLA-4 in the LLC model. Tumor surface in mm2 (mean ± SD) of LLC tumors that were injected on day 0, mice (n = 57) were treated with anti-CTLA-4 and/or gemcitabine or cisplatin. A representative of 3 separate experiments is shown (n = 30). The difference in tumor outgrowth was significantly less for the combination treatment from day 13 on when compared with anti-CTLA-4 alone and from day 18 on when compared with gemcitabine alone (p<0.05). (PDF) [file pone.0061895.s007.pdf]

Figure S8

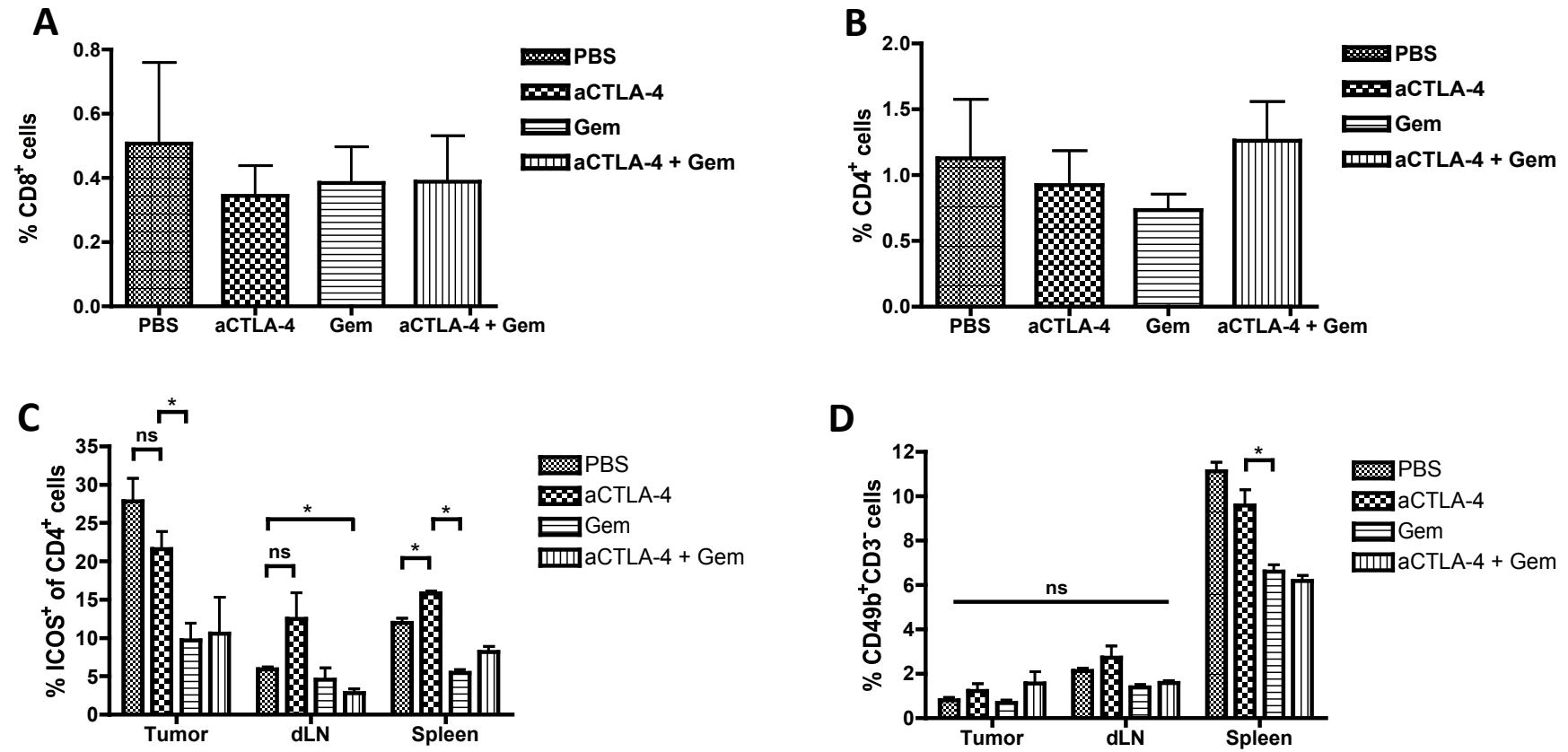

Supplement: Figure S8 — Frequencies of CD4+ Th cells, CD8+ CTLs, CD49b+CD3- NK cells and ICOS+CD4+ activated Th cells in tumor, tumor-draining lymph nodes (TDLN) and spleen. Populations were measured on day 15 (n = 36, 6 mice per group for control and anti-CTLA-4, 12 mice per group for gemcitabine-containing regimes pooled per 2 mice because of the small tumor size in that groups), means with SEMs are shown (*p<0.05). (PDF) [file pone.0061895.s008.pdf]

Figure S9

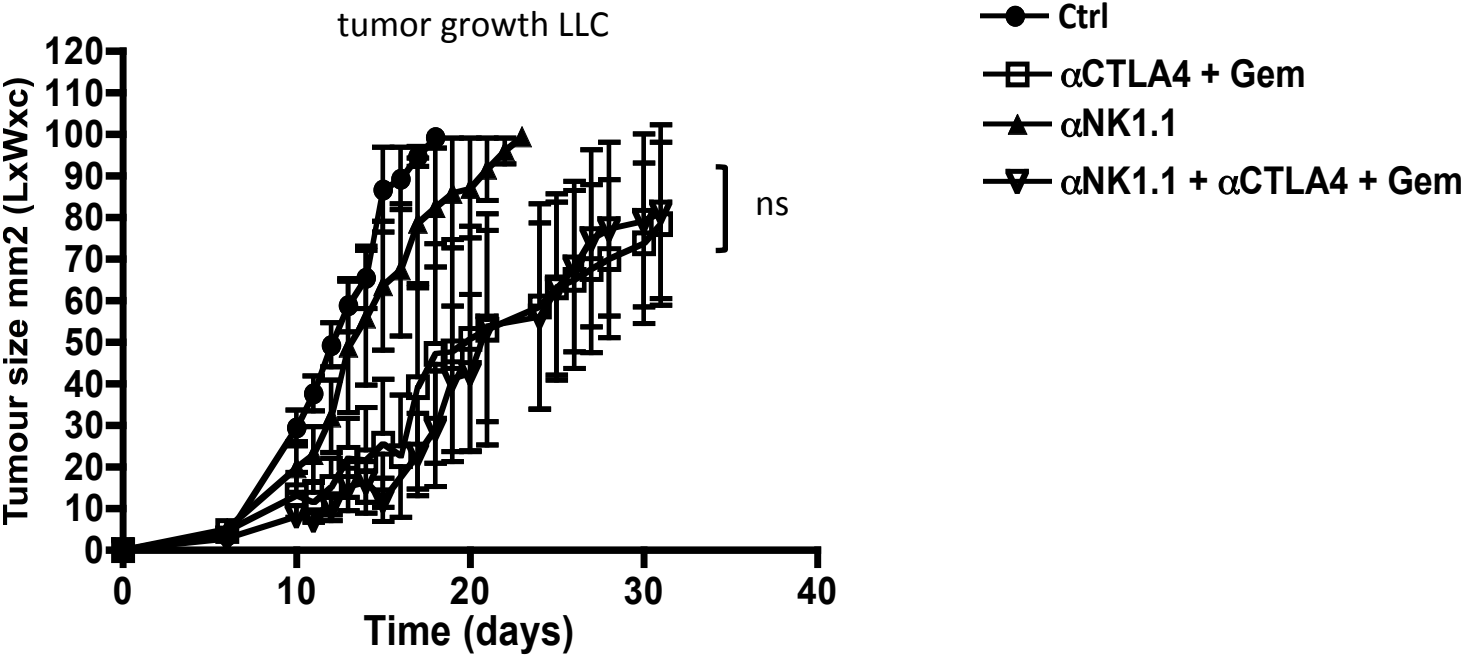

Supplement: Figure S9 — The effect of NK-depletion on the efficacy of gemcitabine and anti-CTLA-4 in the LLC model. Tumor surface in mm2 (mean ± SD) of LLC tumors that were injected on day 0, mice (n = 57) were treated with anti-CTLA-4 and/or gemcitabine in combination with an anti-NK1.1 depleting antibody. A representative of 2 separate experiments is shown (n = 20). Mice were treated with anti-NK1.1 (q3,dx3) starting on day 6 with 150 µg i.v, followed by 200 µg i.p on days 9 and 12. Anti-CTLA4 (q3,dx4) was administered 75 µg i.p on days 9, 12, 15, 18 and gemcitabine (q3,dx5) 120 µg/g i.p on days 9, 12, 15, 18, 21. NK depletion did not change the anti-tumor effect of combination treatment with anti-CTLA-4 and gemcitabine. (PDF) [file pone.0061895.s009.pdf]
